# Assessment of Local Climate Zone Classification Maps of Cities in China and Feasible Refinements

**DOI:** 10.1038/s41598-019-55444-9

**Published:** 2019-12-11

**Authors:** Chao Ren, Meng Cai, Xinwei Li, Lei Zhang, Ran Wang, Yong Xu, Edward Ng

**Affiliations:** 1 Faculty of Architecture, The University of Hong Kong, Hong Kong, China; 2 Institute of Future Cities, The Chinese University of Hong Kong, Hong Kong, China; 3 School of Architecture, The Chinese University of Hong Kong, Hong Kong, China; 4 School of Geographical Sciences, Guangzhou University, Guangzhou, China

**Keywords:** Environmental impact, Socioeconomic scenarios

## Abstract

Local climate zone (LCZ) maps that describe the urban surface structure and cover with consistency and comparability across cities are gaining applications in studies of urban heat waves, sustainable urbanization and urban energy balance. Following the standard World Urban Database and Access Portal Tools (WUDAPT) method, we generated LCZ maps for over 20 individual cities and 3 major economic regions in China. Based on the confusion matrices constructed by manual comparison between the predicted classes and ground truths, we highlight the following: (1) notable variation in overall accuracies (i.e., 60%–89%) among cities were observed, which was mainly due to class incompleteness and distinct proportions of natural landscapes; (2) building classes in selected cities were poorly classified in general, with a mean accuracy of 48%; (3) the sparsely built class (i.e., LCZ 9), which is rare in the selected Chinese cities, had the lowest classification accuracy (32% on average), and the class of low plants had the widest accuracy range. The findings indicate that the standard WUDAPT method alone is insufficient for generating LCZ products that demonstrate practical value, especially for built-up areas in China, and the misclassification is largely caused by the lack of building height data. This result is confirmed by a refinement test, in which the urban DEM retrieved from Sentinel-1 data with radar interferometry technique was used. The study shows a detailed and comprehensive assessment of applying the WUDAPT method in China and a feasible refinement strategy to improve the classification accuracy, especially for the built-up types of LCZ. The study could serve as a useful reference for generating quality-ensured LCZ maps. This study also examines and explores the relationship between socio-economic status and LCZ products, which is essential for further implementations.

## Introduction

Urbanization, as a process that gradually changes the physical landscape, is often accompanied by environmental problems and health challenges, such as urban heat islands, air quality degradation, heat stroke, overweight, hypertension, infection diseases and increasing deaths caused by injuries^1–5^. These challenges are being addressed with advances in urban climatology, where numerical and physical models in combination with urban canopy parameters (UCPs) have been used to study the interaction between city structure and local climate^6–9^. To acquire UCPs, the existing land use and land cover (LULC) data are usually compulsory. However, although several global LULC datasets are available^10–12^, they are inadequate for calculating UCPs because the description of urban morphology is deficient. To fill the data gap globally, the World Urban Database and Access Portal Tools (WUDAPT) project was initiated^13,14^. As a level 0 product, the local climate zone (LCZ) data have been developed to date. The LCZ scheme categorizes the urban surface into 10 built types and 7 natural types^15,16^, from which UCPs can be determined and thereby improved climate model results can be achieved. Due to its detailed description of urban areas, the LCZ scheme is gaining popularity in studies of local air/surface temperature features^16–21^, urban heat islands^22–24^, urban energy budgets^25,26^, outdoor thermal comfort^27,28^, machine learning^20,29^ and urban ventilation simulations^30^. When historical LCZ datasets are available, there are useful for examining the impacts of local cover change on the spatial pattern of land surface temperature^31^, evolution of the climatological effects of urbanization^32^, and urban growth monitoring^33^.

Because the WUDAPT community provides a standard processing workflow for the generation of LCZ data from freely available satellite images (e.g., Landsat and Senitinel-2) and training samples that have been identified according to Google Earth images with open source SAGA software, there is currently abundant LCZ data for more than 120 cities in different continents; these data are being produced and uploaded to the WUDAPT portal after quality assessments^14,20^. In this work, following the standard WUDAPT processing method, we generated LCZ maps for over 20 individual cities and 3 major economic regions in China from Landsat images acquired from 2014 to 2015. The cities vary in size and have distinct geographic locations; additionally, the cities have different levels of economic development. The quality assessment and cross-comparison among these LCZ maps are then rigorously conducted to reveal the factors that limit the accuracy of the WUDAPT method in Chinese cities. To explore the strategies that can improve the accuracy of current LCZ products, we evaluated the role of the urban digital elevation model (DEM) generated from Sentinel-1 data by the synthetic aperture radar interferometry (InSAR) technique. Although SAR data have been used for LCZ classification, only SAR intensity information has been adopted^34–36^, and the role of an InSAR-derived urban DEM for the refinement of LCZ products has not yet been investigated.

The contributions of the work are multi-fold. First, LCZ classification maps with a mean overall accuracy of 76% for more than 50 Chinese cities were generated, which enriched the database of land cover and land use in China and can benefit the study of urban climate^24,37–39^. Second, by mining the confusion matrices of such a large volume of LCZ data, the factors that limit the performance of the default WUDAPT workflow in cities of China have been revealed. Finally, this study sheds light on the directions of further optimization of the WUDAPT method by selecting suitable training samples, considering seasonal discrepancies between training samples and Landsat data, and involving external data sources. Among them, the urban DEM retrieved from freely available Sentinel-1 data shows great potential.

## Results

### Overall accuracies

**Figure 1:**
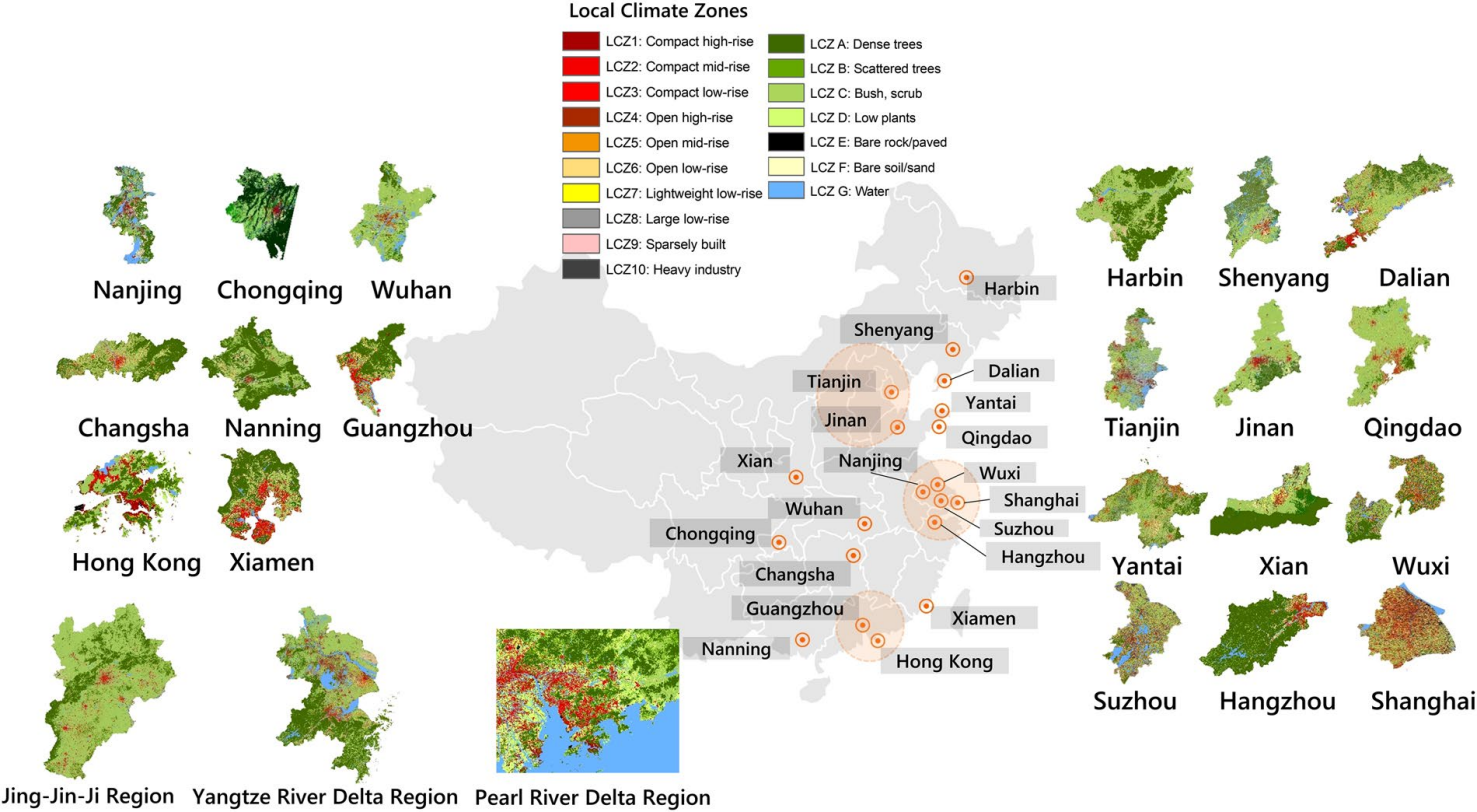
LCZ maps of 20 individual cities and 3 major economic regions.

**Figure 2:**
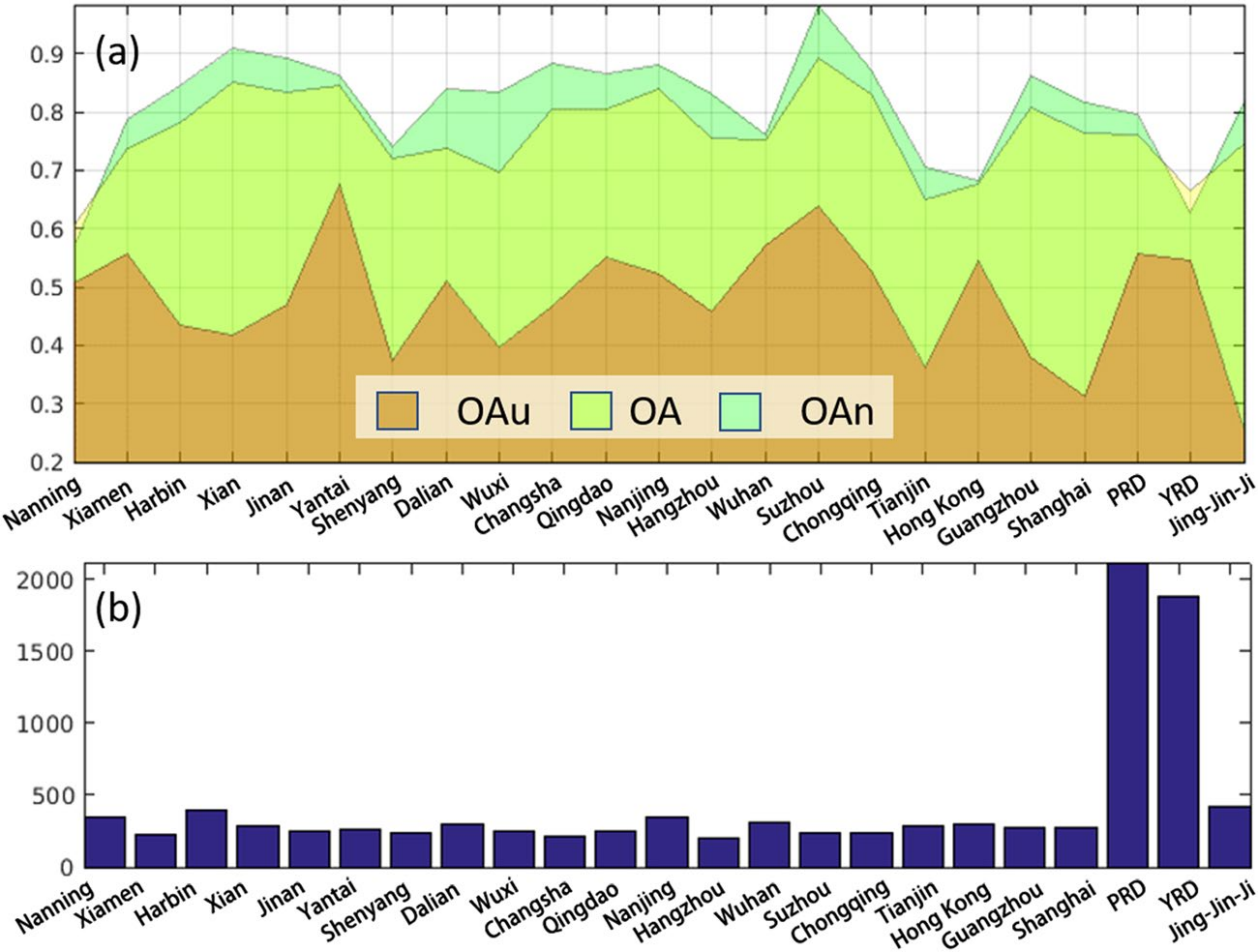
(**a**) Accuracy measures of LCZ maps for selected cities. (OAu: overall accuracy results of built-up classes; OA: overall accuracy results of all classification; OAn: overall accuracy results of natural land cover classes); (**b**) Total number of collected training samples for each study area.

Following the standard WUDAPT processing workflow^40,41^, the LCZ maps of 20 individual cities and 3 major economic regions (i.e., Jing-Jin-Ji, Yangzte River Delta, and Pearl River Delta) were produced (Fig. 1). As many as 15 out of the 20 selected cities can be found on the WUDAPT platform, while 5 cities and 3 economic regions are not yet submitted to the WUDAPT portal at this stage. The quality checking results and the availability of the LCZ map products used in this study are summarized in Table 1. The overall accuracy of these LCZ classification maps ranges from 60% to 89%, with a mean of 76% and a standard deviation of 7.2% (Fig. 2a). The overall accuracy has a similar fluctuation with the accuracy of natural land cover (OA_n_), which has a mean of 85% for the selected cities. Figure 2a also indicates a relatively poor and fluctuating performance of the classifier for built-up areas. The mean accuracy over built-up areas (OA_u_) is 47%, and the lowest is only 26% in the Jing-Jin-Ji region. When compared to the number of training samples shown in Fig. 2(b), there is no statistically significant correlation with these accuracy measures.

**Table 1:** The availability and status of LCZ maps on the WUDAPT platform (in addition to “not available” cities, all other cities can be checked on the WUDAPT website; cities with “minor” or “accepted” status can be downloaded).

WUDAPT Status	Cities				
In preparation	Nanjing	Hong Kong	Xiamen	Suzhou	Wuxi
Accept	Shenyang				
Minor	Wuhan	Changsha	Qingdao	Xian	Shanghai
Major	Jinan	Tianjin	Hangzhou		
Not available	Chongqing	Nanning	Harbin	Dalian	Yantai
	Guangzhou

### Accuracy variation among classes

**Figure 3:**
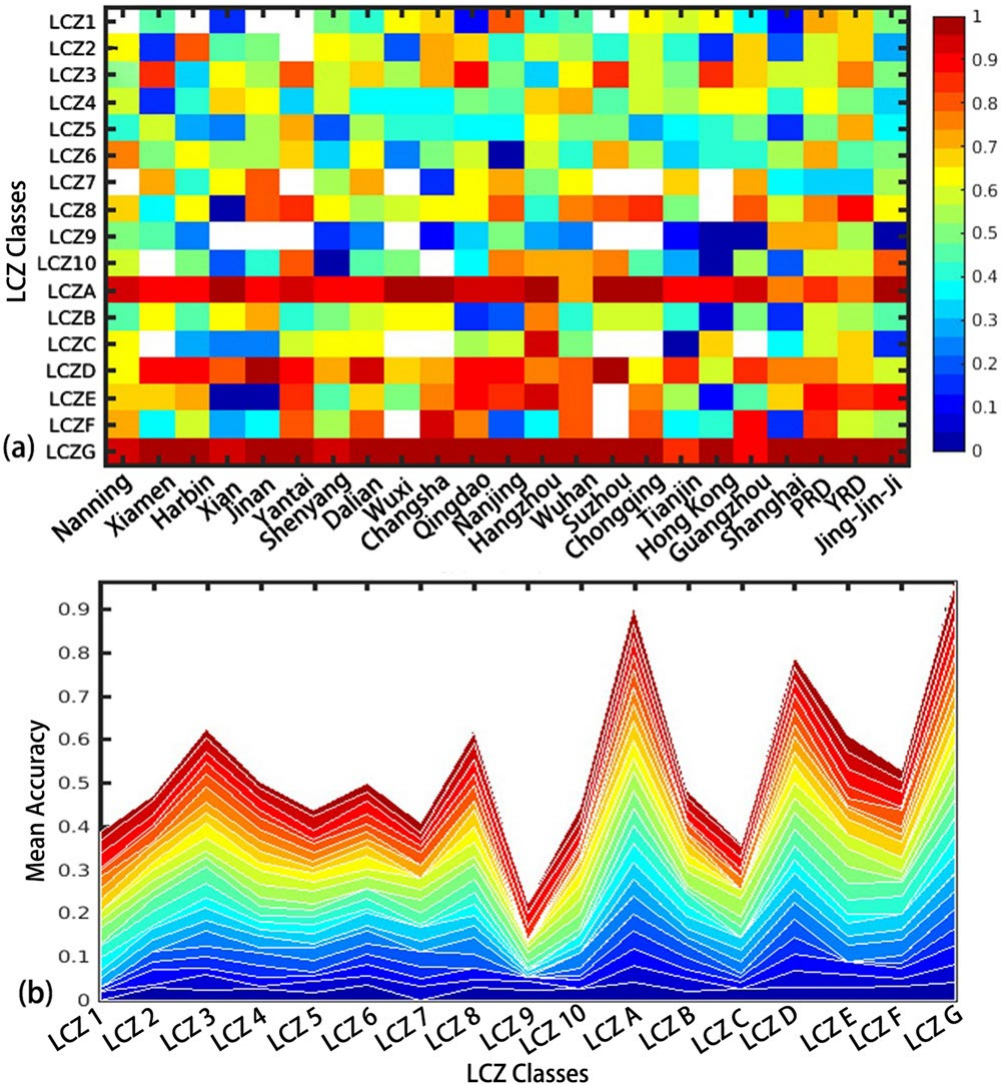
(**a**) The accuracy of each class in all selected cities/regions (X axis shows the name of the cities and regions; Y axis shows the 17 LCZ classifications; the coloured bar shows the LCZ classification accuracy results ranging from 0–1); (**b**) the area map of (**a**) indicating the mean accuracy of each class and the contribution of each city/region (lines represent 20 different cities and 3 economic regions; X axis shows the 17 LCZ classifications; Y axis shows the classification accuracy results ranging from 0–1).

When examining the accuracy matrix of all classes in all cities/regions (Fig. 3a), it is observed that the class of LCZ 9 (sparsely built) has a consistently low accuracy, with a mean of 32% regardless of where the city is located and how developed the city is. It is followed by LCZ C (bush, scrub) and LCZ 5 (open mid-rise), with a mean of approximately 40%. Over built-up areas, serval zero accuracies were observed, mainly appearing in LCZ 6 (open low-rise), LCZ 8 (large low-rise), LCZ 9, and LCZ 10 (heavy industry). On the other hand, three natural LCZ classes (i.e., LCZ A (dense trees), LCZ D (low plants) and LCZ G (water)) were satisfactorily categorized in all cities, with a mean accuracy of 94%, 85% and 95%, respectively. To reflect the contribution of cities to the classification accuracy and discrepancies among these classes, an area map is shown in Fig. 3b. It is observed that, for classes with high accuracy (e.g., LCZ A), the performance of the WUDAPT method in all cities is stable regardless of the variation in the number of training samples, while for the poorly classified LCZs (e.g., LCZ 9), the performance fluctuates notably, indicating that single-city optimization might be needed for the improvement of classifications with low accuracy.

### Confusion matrix

**Figure 4:**
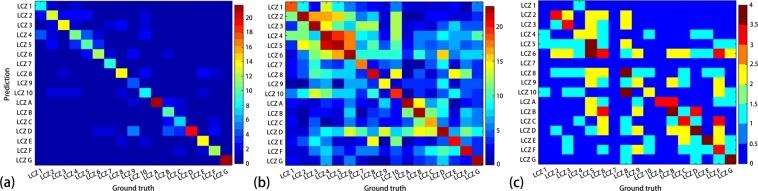
(**a**) Accumulation of normalized confusion matrix where the original elements have been divided by the row total; (**b**) appearance number of each class in all the confusion matrices; (**c**) appearance number of each class in the confusion matrices of Moscow, Warsaw, Yangon and Karachi. The coloured bar shows the number of cities.

By investigating the confusion matrix, the accuracy discrepancy could be explained by misclassification among the LCZ classes. To obtain a general picture of the class mixture over all cities, we accumulate the confusion matrices by summing the corresponding elements (Fig. 4a). It is clear that a large portion of the LCZ classes have misclassification errors. For example, the worst-classified LCZ 9 was mistakenly recognized as LCZ D in 56% of cities. As the land cover of heavy industry (LCZ 9) could be compact/open/large low-rise (LCZ 3, LCZ 6 and LCZ 8, respectively) and possibly contain large paved surfaces (LCZ E), confusion among these classes is common in the selected cities. To better understand how often these classes could be confused, the frequency of each class based on the confusion matrix was counted and the results are shown in Fig. 4b. It can be seen that classes in built areas (i.e., LCZs 1–10) are notably confused, and the pattern is distinct from the similarity matrix used for calculating the weighted accuracy^42^ and from those published^35^. As a comparison, Fig. 4c shows the combined confusion matrix from LCZ maps for Moscow, Warsaw, Yangon and Karachi, which is also distinct from that for Chinese cities. In addition to the urban classes, LCZ D (low plants), a class of natural surfaces, has the widest mixture range. It was mistakenly identified as LCZs 6, 9, B (scattered trees), C and F (bare soil or sand), all of which have a common feature of scattered trees according to their definitions.

### Accuracy variation among cities

**Figure 5:**
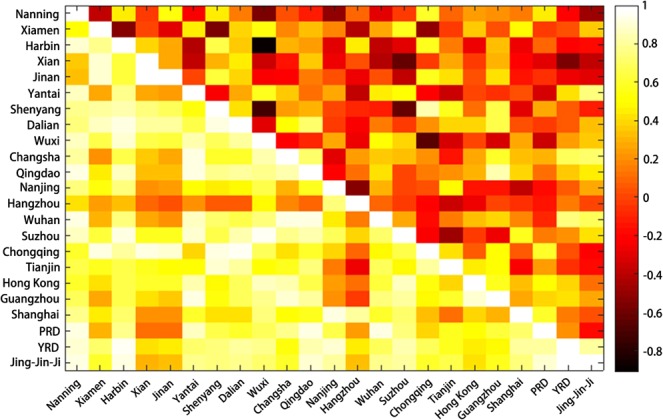
The correlation matrix of classification accuracies among cities/regions. The upper triangular part shows the correlation coefficients of urban areas, and the lower triangular part shows those of natural lands. The coloured bar shows the correlation coefficients ranging from −1 to 1.

When focusing on the quality assessment in each city, it is noticed that approximately half of the cities do not contain all the LCZ classes, among which, the city of Suzhou has the fewest classes (6 less). Poor classifications in the selected cities mainly occur in built-up areas, with exceptions in that Xian, Jian and Tianjin have the worst classifications of natural lands. In these cities, zero accuracy was observed in LCZ E (bare rock or paved) and LCZ C (low plants), where there were misclassifications with LCZ F in Xian, LCZ 10 in Jinan and LCZ B in Tianjin. To investigate the relationship of classification accuracies among cities, following Eq. (2), we calculated the correlation matrix where the upper triangular part represents the correlation coefficients of urban classes and the lower part represents the coefficients of natural classes (Fig. 5). It is clear that the selected cities have comparable accuracies in each class of natural areas, while the correlation of accuracies of built-up classes among cities is generally low regardless of the LCZ type or city size. Considering that the performance of classifiers in natural lands is satisfied, it comes as no surprise that cities with less compact and open built-up classes (i.e., LCZs 1–6) usually have higher overall accuracies, e.g., Suzhou, Nanjing, Yantai, and Xian.

### LCZ map refinement with urban DEM

**Figure 6:**
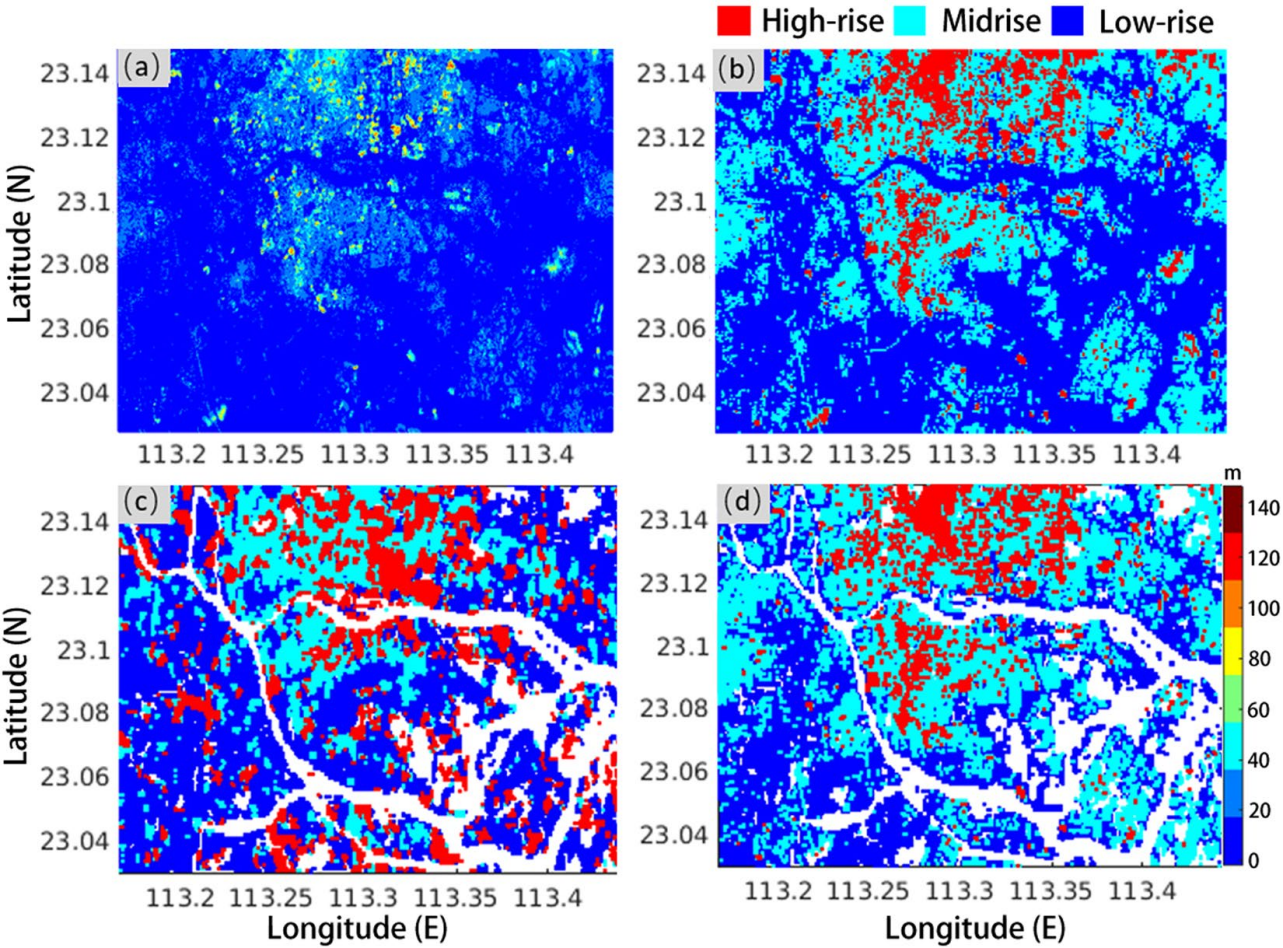
(**a**) Urban DEM retrieved from Sentinel-1 data; (**b**) classifications according to mean heights over 100 m patches; (**c**) height-related classifications extracted from the LCZ map; (**d**) height-related classifications extracted from (**b**).

The analysis of the confusion matrices indicates that the WUDAPT method is insufficient for identifying distinct building classes and that height information is expected to be vital for refinement. There are several techniques to acquire urban elevation data (e.g., LiDAR and photogrammetry); however, the cost is usually prohibitive, especially for large-scale mapping. To improve the accuracy of classifying urban built-up classes and staying in line with the philosophy of WUDAPT, that is, using freely available imagery, we generated the urban DEM with 25 m resolution from C-band Sentinel-1 SAR data using our self-developed TCPInSAR processor to detect the impact of adding then urban DEM into the LCZ scheme. Figure 6(a) shows the elevation map over an area in the PRD region. According to the definitions of the LCZ classes, we use thresholds of 10 m and 30 m to distinguish low-rise, midrise and high-rise classes. The classification map is shown in Fig. 6(b), whose resolution has been down-sampled to 100 m. To make a cross-comparison, we extracted and shrank the LCZ building map into three classes by combining LCZ 1 and LCZ 4 as the high-rise class, LCZ 2 and LCZ 5 as the midrise, and others as the low-rise, as shown in Fig. 6(c). Figure 6(d) shows the corresponding map constructed from the InSAR-derived urban DEM. Significant discrepancies are observed between Fig. 6(c,d), which indicates the uncertainties associated with the current LCZ products. These discrepancies also showed the feasibility of using InSAR-derived DEM products in the refinement of classifying LCZ urban classes.

### Correlation among cities in class distribution

**Figure 7:**
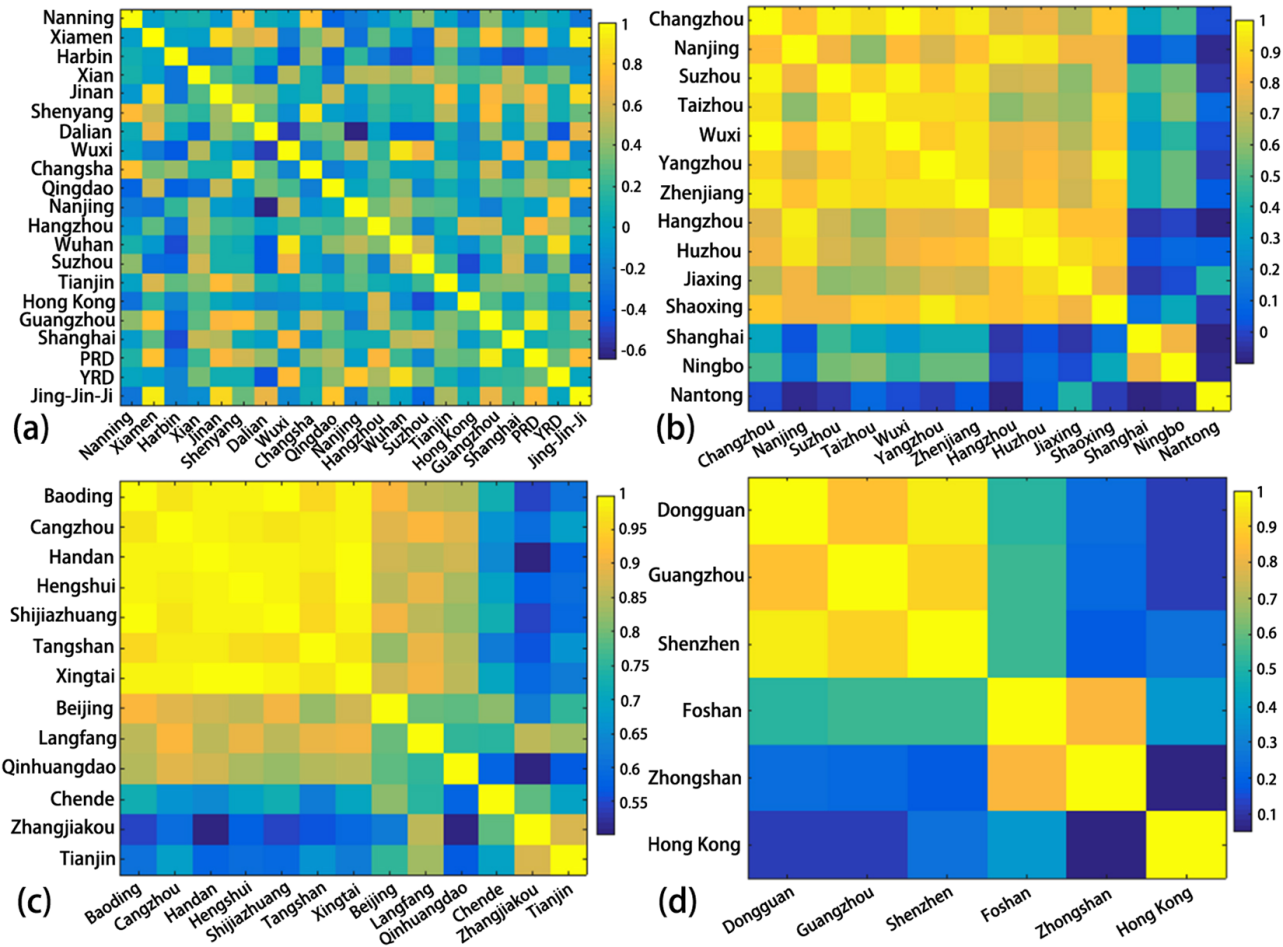
Correlation in class proportions among (**a**) the selected cities/regions; (**b**) cities in the Yangtze River Delta region; (**c**) cities in the Jing-Jin-Ji region; and (**d**) cities in the Pearl River Delta region. The coloured bar shows the correlation coefficients ranging from −1 to 1.

Although the current accuracy of urban classes might not be sufficient to support a comprehensive analysis of the spatial pattern of each single class, it is possible to reveal the similarity among cities and regions with respect to class distribution by correlation analysis. Following Eq. (2), we calculated the correlation coefficient of the proportion of LCZ urban classes among the selected cities, as shown in Fig. 7. In general, the urban structures of cities have a low similarity among all selected cities regardless of city size, geographic location, and social economic status (see Fig. 7(a)), which indicates that unique urban canopy parameters should be determined for each city when applying numerical models to simulate the weather, climate and air quality. However, when looking into the cities within each economic region, the correlation appears to be notable. Cities located in the same economic region tend to have similar proportions of urban LCZ classes. In the Yangtze River Delta region, except for the city of Nantong, the other 13 cities have a high correlation (>0.8) in class proportion with one or more cities (Fig. 7(b)). A higher correlation (>0.9) among most of the cities was also observed in the Jing-Jin-Ji region, where only the cities of Chengde and Zhangjiakou were distinct from the other 11 cities (Fig. 7(c)). The correlation matrix over cities in the Pearl River Delta region is shown in Fig. 7(d), where the class distributions in cities maintain a correlation of at least 0.8 except for that in Hong Kong, which, as expected, as one of the most developed cities in the world, was different from the other cities in the region.

## Discussion

**Figure 8:**
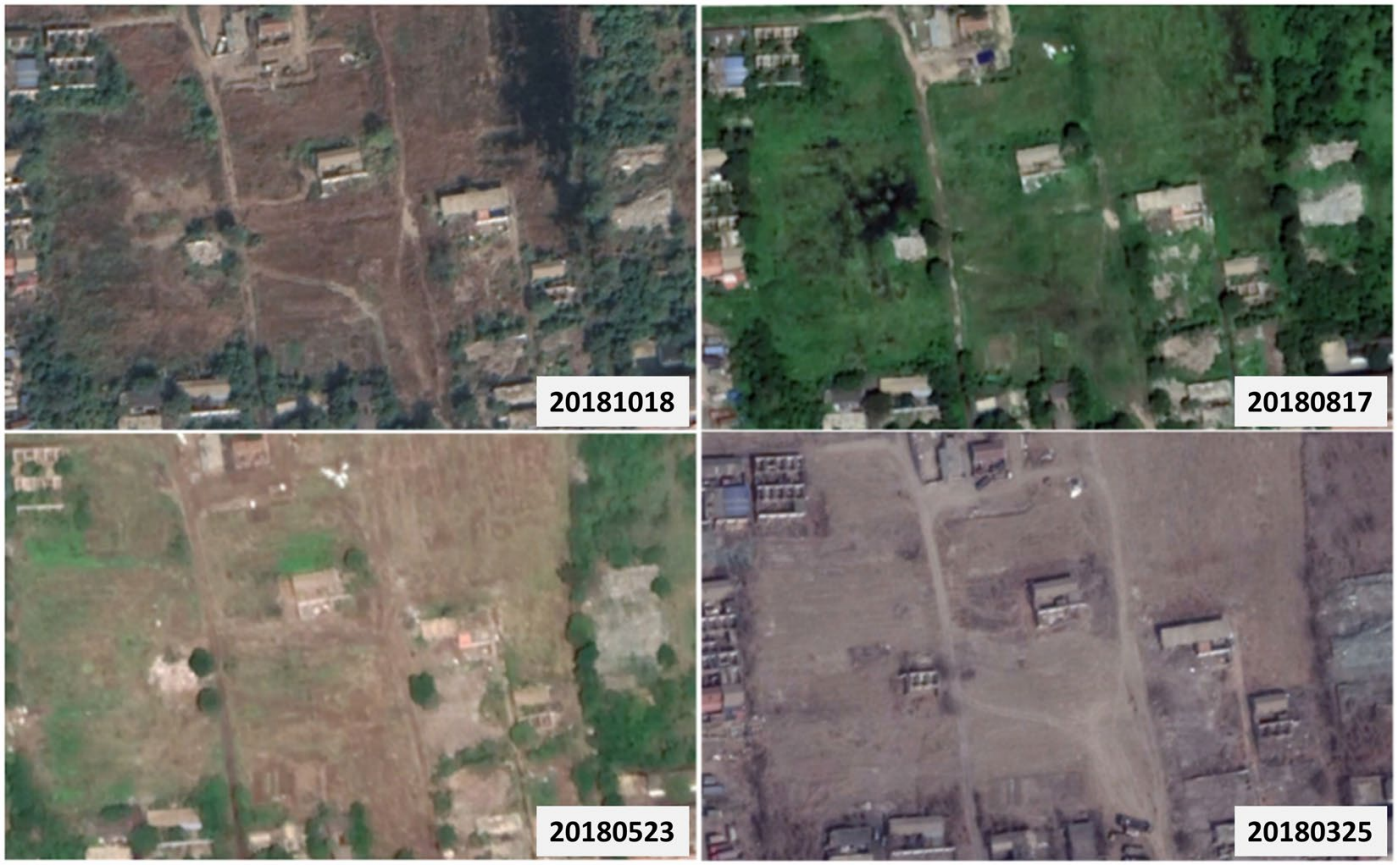
Spatial features of the same training sample in different seasons. The figures were captured from: Google, DigitalGlobe.

The generated LCZ maps have notable variations in classification accuracies (especially for the built-up classes). We attribute this result to the following reasons: (1) class incompleteness. It is commonly accepted that the WUDAPT method has relatively poor classification performance for compact and open buildings (i.e., LCZs 1–6). Therefore, for cities that lack such classes, a higher classification quality can be anticipated. Examples in this work are Yantai and Suzhou. (2) Insufficient training samples. Although the significant relationship between the number of training samples and accuracy was not observed in the selected cities, we noticed that Hong Kong, owning the highest sample density in built-up areas, has the best urban classification performance, while the worst is the Jing-Jin-Ji region, where the sample density is lowest. Using LCZ 9 as another example, most cities performed poorly in this class except for Harbin, where the training samples were rather abundant, up to 28% of the total amount. (3) Similarity among classes. From the generated LCZ maps, it is found that, in addition to the misclassification among compact and open buildings, a large portion of classes were mistakenly recognized as LCZ 10 and LCZ D in the selected cities. Indeed, industrial structures could contain compact or open buildings. (4) Seasonal mismatch. When selecting training samples from Google Earth, seasonal differences with Landsat images deserve considerable attention. It is particularly risky for cities with four distinct seasons. Figure 8 shows the land features of a training sample collected in the Jing-Jin-Ji region in different months, indicating that a seasonal mismatch could exist between the training samples and the Landsat and might cause misclassification.

During the generation of LCZ maps, to improve the efficiency, we jointly processed some cities as a cluster. Among the three major economic regions, the Yangtze River Delta and Pearl River Delta have comparable (or even higher) classification accuracies, while the performance (especially in built-up areas) over Jing-Jin-Ji is relatively poor, mainly due to insufficient samples (only 420 over the whole territory). Such practice confirms that large-scale processing is feasible provided that cities share similar biophysical backgrounds and the features of training samples are consistent.

When looking into the confusion matrices, severe misclassification mainly occurred among urban classes in the selected cities. To distinguish them, information about the relative locations and heights of buildings is vital; however, optical images are insufficient to identify building heights, likely leading to the incorrect selection of training samples and thereby incorrect classifications. Building heights seem to be compulsory for accurate classification of urban landscapes, which was verified by a preliminary experiment with InSAR-derived urban DEM. Although the urban DEM has a moderate resolution of 25 m, it provides more reasonable height-related classifications, indicating that building classifications can be refined with extra height information.

The correlation analysis among cities reveals that similarity in class proportions is regionally dependent. Cities within the same economic region have rather high correlation coefficients, indicating they have a higher similarity in the proportions of different built-up types, while the correlations among all cities are relatively low. The potential causes include similar social, economic and climate status. However, within each region, there are still cities with distinct urban structures, and this situation leads to low correlations. The distinction is mainly raised by one or more classes that have extraordinary proportions. For example, Nantong in the Yangtze River Delta region has 48.1% of sparsely built (i.e., LCZ 9) land covers in urban areas, which is much higher than that observed in other cities. Hong Kong in the Pearl River Delta region is distinct from the others by having 30.6% of the compact high-rise class (i.e., LCZ 1), the highest in the region. The high similarity in LCZ classes among cities within respective regions might indicate that the convergence of city development is serious, which can serve as a reference for regional planning and policy making.

## Conclusion

Following the standard WUDPT processing workflow, the LCZ maps were generated for 20 individual cities and 3 major economic regions in China based on site-specific operation. By examining the difference in classification accuracy, we conclude that the accuracy discrepancy among cities is mainly due to class incompleteness (especially the compact classes), the number and quality of training samples, the similarity among classes and the seasonal mismatches between samples and Landsat data. This result indicates that because Google Earth’s image does not include the height information of buildings, the standard WUDAPT method is inadequate for distinguishing height-related classes. Considering that the similarity among classes often makes the sample collection rather challenging, even for experienced operators, novel classifiers other than supervised ones should be developed. For cities with distinct seasonal land features (especially those in northern China), when collecting training samples, seasonal consistency should be ensured, as low plants (LCZ D) in summer could become bare soil (LCZ F) in winter in these cities.

The experiment with the urban DEM demonstrated a reasonable refinement, which shows that the height information retrieved from satellite SAR images (e.g., Sentinel-1) has the potential for distinguishing and detecting the building heights of LCZ classes 1–10 in built-up areas and can improve the current WUDAPT method and level 0 product accuracy. However, although Sentienl-1 data are freely available around the world, it is challenging for non-InSAR experts to extract the urban DEM from these data. The main difficulty lies in the mitigation of building/ground deformations and turbulent atmospheric delays, which are mixed with building heights in InSAR measurements. Moreover, how to tightly integrate the data in the WUDAPT method (e.g., dataset alignment and height classification threshold selection) also needs further investigation. To facilitate the usage of the urban DEM in LCZ classification, the generation of annually updated national urban elevation datasets from interferometric Sentinel-1 measurements will be a part of our future work, which is also expected to benefit WUDAPT L1 (i.e., 2.5D urban forms) data production.

It is worth noting that, although the building classes are currently of moderate accuracy, i.e., approximately 40%, the potential of LCZ maps can also be explored as they carry more detailed information on urban morphology than do other classification datasets and they are valued at both the domestic and the international levels. At the national level, the development of LCZ maps for 20 individual cities and 3 major economic regions in China can serve as a fundamental database for various applications, such as urban climate investigation^32^, urban energy consumption estimation^26^, urbanization projection and air pollution distribution detection^43^. The correlation analysis results of the developed LCZ data show the morphological characteristics of both built-up areas and natural landscapes of Chinese cities and regions. The convergence of city development within respective economic regions might be useful for evaluating the collaborative/competitive relationship and degree of regional integration. At the international level, the developed database is also a crucial part of the WUDAPT database because approximately half of the training samples and LCZ maps are from this study. The developed database and training samples not only lead to the development of global and regional LCZ maps but also make data fusion and data transferability possible. For example, our data were adopted in the latest global transferability of local climate zone models^44^. Based on our findings, the study also indicates the use of open data to develop an urban DEM for improving the LCZ classification accuracy of built-up areas.

Moreover, when studying the meteorological effects of urbanization, the LCZ maps can be reduced to 3 classes (i.e., urban low-rise, midrise and high-rise) as one of the inputs of the Weather Research and Forecasting (WRF) model to enhance the simulation^32^. Meanwhile, the active WUDAPT community is nurturing innovative methods to produce high-quality LCZ products, e.g., by involving other types of data sources and using advanced machine learning techniques. Accurate and publicly accessible LCZ maps for Chinese cities can be expected in the near future.

## Dataset and Methodology

### Cities

A total of 20 individual cities and 3 major economic regions (i.e., Jing-Jin-Ji, Yangzte River Delta, and Pearl River Delta) were selected for LCZ map generation (Fig. 1). Although a large portion of cities are in the eastern segment of the country, they are representative, as the selected cities range from the first (i.e., megacities) to the second (i.e., large-medium cities) to the third-tier levels (i.e., medium cities). These cities contributed more than 50% to China’s GDP in 2018. Considering the ecoregion similarity^20^, the cities in China’s three major regions are jointly processed.

### Data

The 30 m Landsat 8 level 1 images from 2014 to 2015 were downloaded from the U.S. Geological Survey (http://glovis.usgs.gov). They were used for LCZ map generation for the cities in China. Seamless mosaic and atmospheric correction were conducted if necessary, and all images were down-sampled to 100 m to reflect the local urban structures instead of single objects. For urban DEM generation, a set of Sentinel-1 radar data acquired in 2015 with a spatial resolution of 25 m were collected from the European Space Agency (https://scihub.copernicus.eu/dhus/#/home).

### Training samples

Training data are vital for LCZ classification in the standard WUDAPT workflow. In view of efficiency, it is ideal to classify a large number of cities by using the training data collected in one or a few cities. However, such a strategy usually fails to produce satisfied classification data due to transferability, a common issue faced by machine learning methods, which has also been recently reported^20^. Therefore, in our work, although it is time consuming, we generated the training polygons for each LCZ class in every city/region following the guidance on the WUDAPT website. To distinguish the classes that appear similar in Google Earth images (e.g., compact low-rise (LCZ3), open low-rise (LCZ6) and lightweight low-rise (LCZ7)), Google Street View images were also employed. Considering that the Landsat images were acquired during a two-year period, when selecting training samples, we avoided digitizing polygons in places that were likely to change, such as construction sites, vacant development areas and bare land^19^. We selected a total of 9923 samples from these cities. The number of samples for each city/region is shown in Fig. 2b. It is worth noting that some types of LCZ classes were not available in these cities. To reduce the effect of sample imbalances, we selected a similar number of samples from each class, provided that it was abundant in the cities.

### WUDAPT processing workflow

There are 3 steps involved in the standard WUDAPT processing workflow for the generation of LCZ maps^13–15^: (1) acquisition and pre-processing of freely available satellite images (e.g., Landsat-8); (2) selection of training samples by experienced operators for each city; (3) conduction of supervised classification (e.g., random forest) embedded as an ‘LCZ classification tool’ in SAGA GIS. The workflow can be efficiently applied in each city while considering that a large portion of selected cities are located in the top 3 megacity clusters of China and that they share similar biophysical backgrounds; based on this information, we combined the samples in each cluster and generated the entire LCZ map for the region^45^.

### Quality assessment and correlation coefficients

To evaluate the accuracy of the LCZ maps, a random sampling scheme (i.e., bootstrapping) is used in the WUDAPT workflow^14^, where the training samples are divided into two portions for training and evaluation. Considering that the collection of a sufficient number of samples requires a long period and that only a limited number of samples can be selected for the classes that are rare in a given city, we conduct the quality assessment manually after the generation of LCZ maps using all samples. A new set of samples was randomly collected for each LCZ class as ground truths. The number of ground truths was set to 0.5% of each generated class. By cross-comparison between the ground truths and produced LCZ maps, the confusion matrices can then be determined, and these matrices reflect the misclassification of land covers of each city. To reflect the overall misclassification of all the selected cities/regions, an accumulated confusion matrix was also determined, where the number of evaluation samples was replaced by the percentage to address the ground truth imbalance among cities. Based on the single-city confusion matrix, a set of quality metrics can be calculated. We employed the following accuracies to evaluate the quality of the LCZ maps^14,20^: (1) overall accuracy (OA), which denotes the percentage of correctly classified pixels, regardless of the performance of each class; (2) OA_b_ reflects the overall accuracy of the LCZ classes related only to built-up areas; and (3) OA_n_ represents the overall accuracy of the LCZ classes of natural land covers. They can be calculated as follows:

1 $$OA=\frac{\mathop{\sum }\limits_{i=1}^{17}{N}_{i}^{c}}{{N}^{a}};\,O{A}_{u}=\frac{\mathop{\sum }\limits_{i=1}^{17}{N}_{i}^{cb}}{{N}_{built}^{a}},\,O{A}_{n}=\frac{\mathop{\sum }\limits_{i=1}^{17}{N}_{i}^{cn}}{{N}_{natural}^{a}}$$

where ${N}_{i}^{c}$, ${N}_{i}^{cb}$ and ${N}_{i}^{cn}$ are the correctly classified areas of all classes, built-up classes and natural classes, respectively, and *N*^*a*^ ${N}_{built}^{a}$ and ${N}_{natrual}^{a}$ are the corresponding total ground truths. In addition to the aforementioned accuracy measures, there are two other commonly used metrics, i.e., OA_bu_, which denotes the overall accuracy of the built versus natural LCZ classes, and the weighted accuracy (OA_w_), which is obtained by applying weights to the confusion matrix to account for the similarity and dissimilarity among classes. Considering that these two measures are usually high and could not reflect the discrepancies among cities, we discarded them in this work. To reflect the reliability of the generated LCZ maps (i.e., how likely that the class on the map will actually be present on the ground), we also calculated the user’s accuracy by taking the total number of correct classifications for a particular class and dividing it by the row total. Because a large number of LCZ maps were generated, we attempted to investigate the relationship among the cities by calculating the correlation coefficients that are defined as follows^46^.

2 $${r}_{xy}=\frac{\mathop{\sum }\limits_{j=1}^{n}({x}_{j}-\bar{x})({y}_{j}-\bar{y})}{\sqrt{\mathop{\sum }\limits_{j=1}^{n}{({x}_{j}-\bar{x})}^{2}}\sqrt{\mathop{\sum }\limits_{j=1}^{n}{({y}_{j}-\bar{y})}^{2}}}$$

where n is the number of LCZ classes (i.e., 17); *x*_*j*_ and *y*_*j*_ are the class accuracies or distributions in two cities, respectively.

### Urban DEM generation

The synthetic aperture radar interferometry (InSAR) technique has long been used for DEM generation. Global SRTM DEM^47^ and TanDEM-X DEM^48^ are typical examples. To retrieve the urban DEM from Sentinel-1 data acquired with a repeating cycle of 12 days, it is compulsory to mitigate the contribution of ground/building deformation and atmospheric artefacts in InSAR measurements. To this end, we adopted our self-developed multi-temporal InSAR technique termed TCPInSAR^49^ to process the data. The estimator has several advanced features, e.g., robust image coregistration^50^, adaptive coherent point selection^44^, quad-tree model for atmospheric delay mitigation^51^ and parameter estimation with no need for phase unwrapping^52^, which guarantees the quality of the retrieved DEM.

## Supplementary information

Supplementary Table S1

## Data Availability

The Landsat data are publicly available here: http://glovis.usgs.gov.The Sentinel-1 data are also publicly available from https://scihub.copernicus.eu/dhus/#/home. For the WUDAPT product, only those after the quality checking are publicly available from: http://www.wudapt.org/.
