# Supplementary material for: Assessment of Local Climate Zone Classification Maps of Cities in China and Feasible Refinements
Source: Sci Rep. 2019 Dec 11;9:18848. doi: 10.1038/s41598-019-55444-9 (PMC6906403; doi:10.1038/s41598-019-55444-9)
Supplement: Supplementary file 1 — Supplementary Table S1 [file 41598_2019_55444_MOESM1_ESM.docx]

**Assessment of Local Climate Zone Classification Maps of Cities in China and Feasible Refinements**

Chao Ren^1,2^, Meng Cai^3^, Xinwei Li^1*^, Lei Zhang^1^, Ran Wang^3^, Yong Xu^4^ and Edward Ng^3^

1. Faculty of Architecture, The University of Hong Kong, Hong Kong China

2. Institute of Future Cities, The Chinese University of Hong Kong, Hong Kong, China

3. School of Architecture, The Chinese University of Hong Kong, Hong Kong, China

4. School of Geographical Sciences, Guangzhou University, China

* Corresponding Author: lixinwei@hku.hk

Supplementary Table S1. The accuracy and the status on the WUDAPT platform of selected 20 cities and 3 regions

|  | WUDAPT status | Overall Accuracy | Built-up Accuracy | Nature Accuracy | Kappa coefficient |
| --- | --- | --- | --- | --- | --- |
| Nanning | N/A | 60.6% | 50.7% | 57.1% | 0.576 |
| Xiamen | In preparation | 73.8% | 55.8% | 78.6% | 0.698 |
| Harbin | N/A | 78.1% | 43.5% | 84.4% | 0.686 |
| Xian | Minor | 85.1% | 41.7% | 90.8% | 0.719 |
| Jinan | Major | 83.2% | 47.0% | 89.2% | 0.787 |
| Yantai | N/A | 84.6% | 67.5% | 86.2% | 0.813 |
| Shenyang | Accept | 71.9% | 37.4% | 74.1% | 0.652 |
| Dalian | N/A | 73.8% | 51.0% | 84.0% | 0.700 |
| Wuxi | In preparation | 69.8% | 39.5% | 83.4% | 0.633 |
| Changsha | Minor | 80.4% | 46.7% | 88.4% | 0.770 |
| Qingdao | Minor | 80.5% | 55.0% | 86.5% | 0.768 |
| Nanjing | In preparation | 84.0% | 52.3% | 88.1% | 0.790 |
| Hangzhou | Major | 75.5% | 45.9% | 83.2% | 0.722 |
| Wuhan | Minor | 75.2% | 57.2% | 76.2% | 0.701 |
| Suzhou | In preparation | 89.3% | 63.9% | 98.4% | 0.842 |
| Chongqing | N/A | 83.1% | 52.8% | 87.1% | 0.777 |
| Tianjin | Major | 64.9% | 36.1% | 70.6% | 0.586 |
| Hong Kong | In preparation | 67.7% | 54.4% | 68.2% | 0.648 |
| Guangzhou | N/A | 80.6% | 38.1% | 86.2% | 0.728 |
| Shanghai | Minor | 76.3% | 31.3% | 81.7% | 0.662 |
| PRD Region | N/A | 76.0% | 55.8% | 79.4% | 0.731 |
| YRD Region | N/A | 66.5% | 54.6% | 62.8% | 0.637 |
| Jing-Jin-Ji Region | N/A | 74.6% | 25.5% | 81.8% | 0.656 |
